# Infrastructure as Software in Micro Clouds at the Edge

**DOI:** 10.3390/s21217001

**Published:** 2021-10-22

**Authors:** Miloš Simić, Goran Sladić, Miroslav Zarić, Branko Markoski

**Affiliations:** 1 Faculty of Technical Sciences, University of Novi Sad, Trg D. Obradovića 6, 21000 Novi Sad, Serbia; sladicg@uns.ac.rs (G.S.); miroslavzaric@uns.ac.rs (M.Z.); 2 Technical Faculty Mihajlno Pupin, University of Novi Sad, Đure Đakovića bb, 23000 Zrenjanin, Serbia; markoni@uns.ac.rs

**Keywords:** distributed systems, cloud computing, edge computing, infrastructure as software, infrastructure as code, micro clouds, micro data centers

## Abstract

Edge computing offers cloud services closer to data sources and end-users, making the foundation for novel applications. The infrastructure deployment is taking off, bringing new challenges: how to use geo-distribution properly, or harness the advantages of having resources at a specific location? New real-time applications require multi-tier infrastructure, preferably doing data preprocessing locally, but using the cloud for heavy workloads. We present a model, able to organize geo-distributed nodes into micro clouds dynamically, allowing resource reorganization to best serve population needs. Such elasticity is achieved by relying on cloud organization principles, adapted for a different environment. The desired state is specified descriptively, and the system handles the rest. As such, infrastructure is abstracted to the software level, thus enabling “infrastructure as software” at the edge. We argue about blending the proposed model into existing tools, allowing cloud providers to offer future micro clouds as a service.

## 1. Introduction

Currently, one of the biggest challenges the IT industry is facing is maintaining uptime. Providing uninterrupted network connectivity and infrastructure availability has become a cornerstone of successful businesses, and a crucial requirement for cloud providers. It is a significant problem for the areas with dynamic networking (e.g., edge computing) especially. Micro and nano Data Centers (DCs) at the edge of the network are taking off [1], and efficient management and configuration of the dynamic networks is a challenge [2].

Elliot et al. estimates that downtime costs between 100k USD, up to staggeringly 1 m USD per hour, for critical application failures [3,4]. A key factor that causes downtime is configuration drift. Phelps et al. reported that drift in configuration is responsible for over 40% of total experienced downtime [5].

Configuration drift represents a state, where the systems become different over time, while it should remain absolutely identical [6]. This drift happens during upgrades or changes that are executed partially. Partial failures are dangerous, because different components may be affected through a cascading effect, or the system may end up in a non-consistent state having different behaviour for two identical actions.

In the era of cloud computing, the Internet of Things (IoT), edge computing, and other cyber-physical systems, continuous delivery, microservices, DevOps are growing rapidly. The infrastructure needs to be constantly deployed and maintained, so it would be beneficial to view the infrastructure as software (IaS) [7]. The benefit of this approach lies in the already available tools, principles, and techniques (e.g., reuse, testing, modeling, and evaluation) that can equally be used for the infrastructure definitions [7,8].

The cloud infrastructure is already abstracted by the software, due to the ever increasing demands [9]. This allows practices like DevOps [10] to facilitate agile processes [11], and launch infrastructure into production environments within seconds. Similar techniques could be used in contexts where cloud computing is intertwined with edge computing bringing cloud principles close to the ground, to form new human-centered applications.

Small data centers deployment at the edge of the network are becoming more popular in recent years [1]. Geo-distributed small-scale servers introduced by edge computing, with heterogeneous resources organized locally as micro clouds (μCs), community clouds, or edge clouds [12] offer interesting opportunities for the future. The infrastructure should be defined separately from the physical machine and operating system, allowing us to have an infrastructure definition that is versioned, automated, and applied repeatedly and consistently every time, hence minimizing configuration drift. This allows better utilization and organization of resources μCs that exist closer to the users, serving requests locally first, and only contacting the cloud if and when needed, increasing the quality of service (QoS).

Ryden et al., suggest that these small-scale servers can help power-hungry servers reduce traffic [13], sending to the cloud only crucial information for the services and/or applications, and not ingesting everything as the traditional cloud computing (CC) model advises. On the other hand, in such a multi-tier infrastructure that spans over clouds and edge computing nodes in a geo-distributed environment existing tools lack support [1,14] for abstracting infrastructure as software.

The deployment of geo-distributed infrastructure is a complicated and challenging process [15], and the key problem that needs to be resolved is how to simplify micro data center (μDC) formation and management [16]. The naive approach would require going to every node and/or cluster and doing it manually. This process is tedious and time-consuming, especially in a geo-distributed environment.

Resources at the edge are distributed by local population needs [3], but in some cases, resources do not have the same distribution in a geo-distributed context (e.g., a new catastrophic event happens in some area, and we need more resources there to support emergency response activities). Some resources might be more important in one place, while others need to be shared across multiple places to control the latency, scalability, and availability [1,3,4].

This needs to be handled descriptively, dynamically and ad hoc [1], since users cannot know and predict all scenarios in advance. A fine-grained control per resource and infrastructure is mandatory property, and could be potentially achieved if we abstract μDCs infrastructure as software.

In this paper, we propose an IaS solution influenced by the existing Infrastructure as a code (IaC) solutions used in the cloud to automate the configuration and provisioning process of infrastructure using cloud instances [17], with adaptations for a different environment. The proposed model can set up the infrastructure of the geo-distributed μCs at the edge dynamically, and provide the ability to manage them properly. Here, geo-distribution means in proximity to some large populations, and μCs are formed, serving their requests locally first. The newly formed model will expand peer-to-peer systems into new directions and blend them with the cloud, allowing novel human-centered, cloud-like applications to be created.

Our approach may be viewed as a transient solution that tries to primarily allow reorganization of local resources into dynamical, better utilized, μCs, before resorting to cloud resource allocation for more complex tasks.

Some existing solutions (e.g., Kubernetes) go as far as treating clusters as disposable—“treating clusters as cattle, not pets” (i.e., numerous servers/clusters built using automated tools designed for failure, where no servers/clusters are irreplaceable [18]).

Solution that we propose in this paper goes one step further, proposing the creation of disposable μCs dynamically, abstracting infrastructure to the level of software—infrastructure as software. These μCs are designed for failure, using automated tools where no μC is irreplaceable—“treating μCs as cattle, not pets”. This allows users more dimensions to operate and optimize their infrastructure. As a result, resources are extended beyond the single node or cluster allowing more data to be processed or stored closer to the user.

The main contributions of the paper are as follows:

- Simplify the deployment of geo-distributed infrastructure, allowing dynamical formation and management of disposable μCs, closer to the users.
- The possibility for application developers to venture into the “infrastructure programming”, allowing infrastructure to be managed in a similar way as the software is.
- Build numerous μCs designed for failure using automated tools where no μCs are irreplaceable—“treating μCs as cattle, not pets”.

The proposed model could exist as a stand-alone solution, integrated into existing tools (e.g., orchestrator engines), or be an integral element of every cloud provider infrastructure and offered as a service [19].

The rest of the paper is organized as follows: Section 2 discusses related work. Section 3 presents the design and architecture of the system. Section 4 outlines various deployment properties used today and the possibility for dynamical formation of micro cloud infrastructure at the edge. Section 5 presents a case study for the proposed model, and comparison with similar models. Section 6 collects some concluding remarks and future directions of our research.

## 2. Related Work

This section presents the relevant studies of the literature and existing tools relevant to this paper. The related work is summarized in three parts: (1) infrastructure management, (2) the nodes organization at the edge, and (3) advanced Infrastructure tools.

### 2.1. Infrastructure Management

In the era of distributed systems, cloud computing, and microservices the open-source community and different companies provided various tools for the purpose of abstracting infrastructure at the level of software. These tools can be separated into two subgroups, based on how users send instructions to the systems [20] on (1) declarative, and (2) imperative.

Newly created tools like Terraform, Polumni, or CFEngine are representative of the declarative movement, using platform-independent language to specify configuration, policies, security, and much more. The users do not specify explicit commands that the system needs to execute. Instead, they declare what they want to achieve, and leave it for the system to determine the optimal way of achieving it. These tools are turning out to be very important in a multi-cloud environment. The users need to specify artifacts, independently from the cloud provider, and let the system deal with the cloud provider specifics. Every major cloud provider offers a proprietary solution, deeply integrated into their ecosystem.

On the other hand, already existing and well-known tools like Chef, Ansible, and Puppet usually rely on some specific language, and the user needs to code the instructions that must be done to achieve the same or similar job. This is more prone to error, since users may introduce a bug in the system that might be hard to debug and find. At the same time, these tools have existed for a long time, and there are existing best practices and a lot of available examples for users to utilize.

Declarative and imperative tools lack native support for the geo-distributed μCs at the edge. Through some form of extension (e.g., plugin system, service calls, etc.), we can adapt these solutions to work in μC environments. Declarative tools are developed with the specific goal—to set up the cloud infrastructure. Their internal structure needs to be changed to support the geo-distribution of μCs that is somewhat different than traditional cloud infrastructure. Imperative tools might be easier to adapt because their internal structure does not need to be changed. We issue commands and let the running agent execute them in some order. This strategy might introduce unnecessary complications to the system leaving it in a non-consistent state if commands fail.

Our solution is tailored for the μC environment, allowing integration with existing tools or cloud providers to organize geo-distributed nodes into μCs. With this strategy, we are getting the best of both worlds, existing tools set up the cloud infrastructure, while our solution sets up μC infrastructure. The new user application model should be based on the existing cloud application model enhanced with local processing applications, offering users a fast and elegant way to develop new human-centered applications.

### 2.2. Nodes Organization at the Edge

Greenberg et al. [21] introduces the idea of μDCs that operate in a proximity to a big population. Because of their strategic position, they can minimize the costs and the latency for end-users, compared to traditional CC data centers [21]. The minimum size of the μDCs is defined only by the local population needs [21,22], as such, they are reducing fixed costs of traditional DCs. The main feature of μDCs is agility, and the authors describe agility as μDCs ability to dynamically grow and shrink resources to satisfy the resource demands and usage from the most optimal location [21]. The model presented by Greenberg et al. [21], represents a good starting point for building μDCs of various heterogeneous edge nodes in a geo-distributed environment. To achieve more availability we need a few more layers. As an inspiration, we can look at how cloud computing is defined and organized.

Content delivery networks (CDN) in centralized delivery models like CC have bad scalability, as Kurniawan et al. [23] argue in their research. To overcome these centralized problems and bad scalability, the authors proposed a different solution, a decentralized solution. To achieve such tasks, authors were using a network of gateways equipped with some storage as well, for internet services at home [23] forming even smaller DCs—nano DCs (*n*DCs). Authors present a possible usage for these *n*DCs in some large scale applications with much less energy consumption than traditional DCs.

These studies show different options for organizing nodes into smaller data centers closer to the users. As such, they present potential for future research serving user requests locally if possible. Compared to these studies, our model offers users to dynamically create μCs where size is determined only by local population needs, allowing the inclusion of volunteer nodes, if required to support the ever-growing number of requests.

### 2.3. Advanced Infrastructure Tools

Osmotic computing is a new paradigm that aims to decompose applications into microservices and perform dynamic tailoring of microservices in smart environments exploiting resources in edge and cloud infrastructures [2]. The applications developed for osmotic computing should be deployed opportunistically in cloud and edge systems, equalizing the microservices concentrations on the two sides. Our proposed solution can also fit well to the osmotic computing concept, allowing dynamic and efficient management of μC infrastructure to avoid application breakdown and degradation of QoS. This is achieved with edge datacenter configuration support.

Eco Multi Cloud [24] is the hierarchical manager that aims to improve a multi-site data center workload. It is composed of two layers. The upper layer assigns/migrates the workload among remote sites, while the lower layer assigns Virtual Machines to physical hosts within every local site. This approach is flexible and can be utilized to achieve and balance different goals (e.g., reductions of costs, consumed energy, carbon emissions, load balancing, etc.).

The previous studies lack dynamic organization and configuration support for geo-distributed μCs at the edge. They offer a valuable solution for other aspects important in μCs (e.g., efficient balancing between different goals, equalizing the microservices concentrations in the cloud and the edge, constant security monitoring of infrastructure, programmable networks). Our study focuses on the dynamic organization pools of resources in the form of disposable geo-distributed μCs in proximity to users, rather than load management or application management. As such it could be used as a base layer for previous studies.

## 3. Micro Clouds at the Edge

This section explains our model of μCs at the edge, influenced by the traditional CC model but adapted for different environments. It also presents the conceptual model for the μCs at the edge.

### 3.1. High Level Micro Cloud Infrastructure

There exist a few definitions of edge computing today. The traditional description is that the EC is the distributed computing model, implementing processing and storage closer to the data sources [14,22,25]. This ordinal concept can be extended with already familiar models like CC to help power-hungry servers reduce traffic. At the same time, more information ca be processed in proximity to the users, increasing QoS, availability, and reliability of offered services.

Vogels et al. define cloud computing as the aggregation of computing resources as a utility and software as a service [26]. And if we take a look at CC architecture, it consists of three main building blocks: (1) cluster, (2) region, and (3) availability zones [27] allowing users to have available software as a service.

Our solution is based on the previous models, extending them into new directions, and creating a geo-distributed μC model, giving users the unique ability to dynamically form, add or remove pools of resources as needed. Compared to the existing edge computing models, the idea of geo-distributed μCs allows users to expand processing/storage resources beyond a single node or group of nodes into regions of clusters similar to the cloud data center. This aggregated pool of resources allows more data to be processed and stored locally before contacting the cloud. This increases QoS, availability, and reliability of services offered to the users.

We can use a familiar model with adaptations, if we observe μCs as geo-distributed systems, reusing established strategies adapted for the different use-case and environments. As geo-distributed μCs, we can think of computing systems that resemble traditional CC data centers organization, adapted for different environments. These systems will operate on arbitrarily vast geographic areas but near the users, handling their requests before the cloud, and contacting the cloud only when necessary (e.g., no cached data in near μCs, data that needs to be processed exceeds the μCs resources, etc.).

Multiple μC clusters form the next logical concept—region. A region increases the availability and reliability of both the system and applications. A region in a CC and the μC model does not represent the same thing. In the traditional CC model, the region is a physical element of the existing infrastructure [27]. In the μC model, a region could be viewed not as a physical but rather as a logical element. In a μC environment, a concept of region describes a set of clusters (that could be) scattered over an arbitrary geographic region. Regions are fully capable of accepting/releasing clusters, as clusters can accept/release nodes.

In MDCs, a cluster is as big as the population nearby requires [21]. In our solution, we imply this valuable restriction. Combining with the previous definition, we get that the μC regions are composed of at least one cluster but can be composed of much more, to achieve a more resilient, scalable, and available system. Multiple regions form the highest logical concept–topology. Topology must have at least one region, but it could span over multiple regions—the topology is capable of accepting/releasing regions.

When creating clusters, regions, and topologies, the vast distances may introduce huge latency in the system, and should be avoided (in normal circumstances). If there are no free nodes/clusters/regions near to be integrated into one of the concepts, we can choose the next available that might be further away.

Compared to the traditional CC model, where new nodes have to be brought and connected physically to the rest of the infrastructure [28], in the μC model, a node needs to connect to the network. The formation of the higher concepts should be done descriptively and dynamically by changing the concept definition. This rule applies to nodes belonging to the cluster, cluster belonging to the region, and region belonging to the topology. It is important to note that we assume nodes inside the cluster run some membership protocol. Table 1 sums up the difference between cloud and μC infrastructure elements. The accent in comparison is on the physical and the logical infrastructure concepts.

With these simple, yet powerful abstractions, arbitrarily vast geographic areas can be covered, with the ability to shrink or expand clusters, regions, and topologies as needed, forming new pools of resources for applications to utilize. In osmotic computing [2] for example, this can be especially useful (e.g., In cases where applications are shifted in between the cloud and the edge because we can dynamically create similar infrastructure like the cloud and assign a pool of resources needed ad-hoc). The osmotic computing framework does not need to take care of the infrastructure, but only to shift applications to the best possible resource pool.

The organization of these concepts should be optional. We could fit clusters in an interval between *n*DCs [23], and μCs [21], or as wide as the whole city or as small as all devices in a single household and everything in between. Formally, the size of some μC cluster (μCc) can be represented like:

(1) $$\mu Cc\in \left[nDCs,\;\mu DCs\right]$$

Any geographical region can be easily covered, with these simple abstractions, with the ability to shrink or expand clusters, regions, and topologies. Their size should be a matter of need, agreement, and usage of the nearby population.

These μCs have fewer resources, compared to traditional clouds, but their vicinity to the users means they have a much faster response. As resources are limited, in case of a storage, rarely accessed data may be transferred to the cloud, so it may happen that it is not present at μC location at the time of user request, but can be retrieved from the cloud and cache it for later. Formally, the position of μCs is in between tradisional CC and EC like:

(2) $$\mu Cs\in \left(Cloud\;computing,\;Edge\;computing\right)$$

Exclusiveness in the previous formula, means that the position of μCs is moveable in between CC and EC depending on the wanted model, more CC-like or more EC-like, but it should not become either of them. It could be an integral part of both of these models (even at the same time), creating a multi cloud environment [29].

To achieve such elasticity, we must abstract the infrastructure to the level of software, venturing into “infrastructure programming” [7], allowing μC infrastructure to be managed similarly as the software is. This approach allows reusability of the available tools, principles, and techniques (e.g., testing, modeling, and evaluation) [30] that can equally be used for our model.

**Figure 1:**
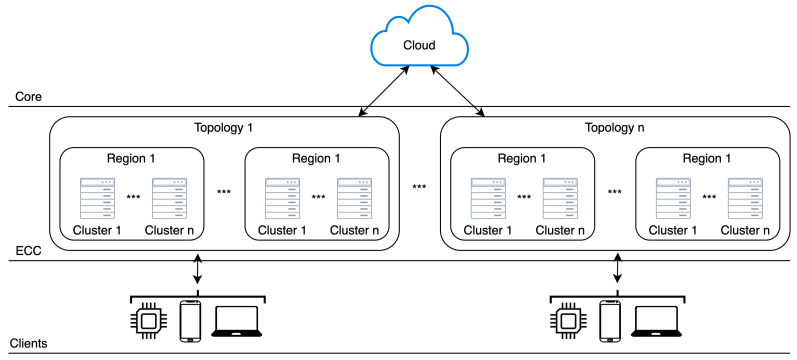
Micro clouds high level architecture model.

Traditional clouds, μCs, and various data sources form a multi-tier architecture, where μCs should be in between traditional clouds and various data sources. Figure 1 presents multi-tier architecture composed of μCs, traditional clouds and various clients—data sources and service consumers.

### 3.2. Conceptual Model of Micro Clouds

A multi-tier computing as briefly described in the previous section plays a crucial role in the delivery of low-latency and data-intensive applications. This model requires flexible infrastructure, so that it can easily be repurposed, essentially reprogrammed, to provide new capabilities [1]. Therefore, the systems must be designed to provide efficient infrastructure management, since these μCs are more diverse than conventional clouds.This section describes a multi-tier infrastructure in greater detail.

On lower levels of multi-tier architecture, response time is the fastest, since data is processed closer to the source. At the same time, there is a limited storage capacity and processing power.

As we go on to the upper tiers, there is more and more storage capacity and processing power available, but the response time is longer, especially when huge volumes of data need to be transferred over the network over a long distance [31].

**Figure 2:**
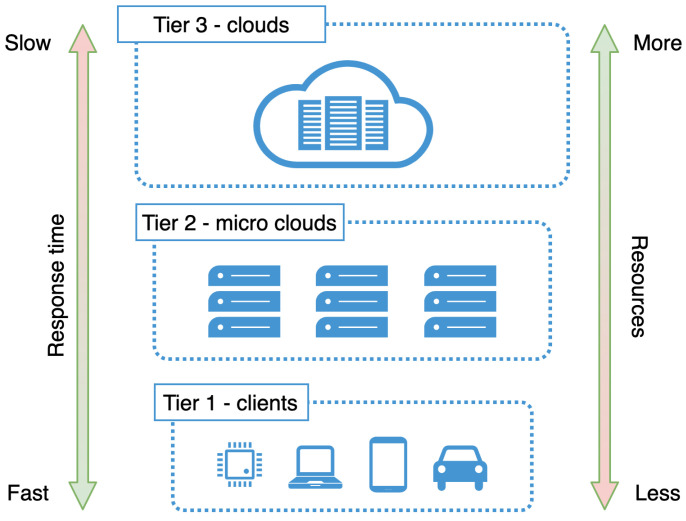
Three-tier architecture, with the response time and resource availability.

Figure 2 shows the three-tier architecture, with the response time and resource availability.

Applications running in these geo-distributed μDCs should serve local requests first—frontend services, but they should also be able to share data to the cloud, on-demand—backend services. This idea would minimize user-perceived latency and increase robustness [1,7], allowing data preprocessing before sending it to the cloud to only transfer data that is important and contains value.

This will allow future human-centered applications to rely more on the data locality principle (i.e., moving the computation closer to the data, instead of moving data to computation [32]), rather than moving huge volumes of data over the network to the cloud for computation, if it is not necessary. This simple idea minimizes network congestion and increases the overall throughput of the system, allowing the computation and storage resources to be deployed at the edge of the network [33] at the best possible location—in proximity to the user issuing the request.

The operators should be able to specify the cluster, region, and topology declaratively. Declarative specifications offer an interesting solution because they hide implementation and deployment details from the user. This will allow us to separate concerns for different types of users [34]. This separation has been widely adopted in cloud computing where a strict separation between development and DevOps teams exists [35,36].

Our model is heavily based on cloud organization, and to implement a tool that will be able to organize and reorganize μCs dynamically, we can take a look at existing IaC and orchestration tools used in the CC today as an inspiration. Tools like Kubernetes, Terraform, and CSBAuditor rely on the reconciler pattern [37].

This pattern enables tracking of resources using two simple states: (1) expected state, and (2) current state. The expected state represents the desired state, while the current state refers to the actual system state. The reconciler pattern runs a reconciliation loop that ensures that the current state remains the same as the expected state.

This means that every node must provide its current state regularly, and when some state is divergent from the desired state, the system must act to ensure that this situation is corrected automatically.

**Figure 3:**
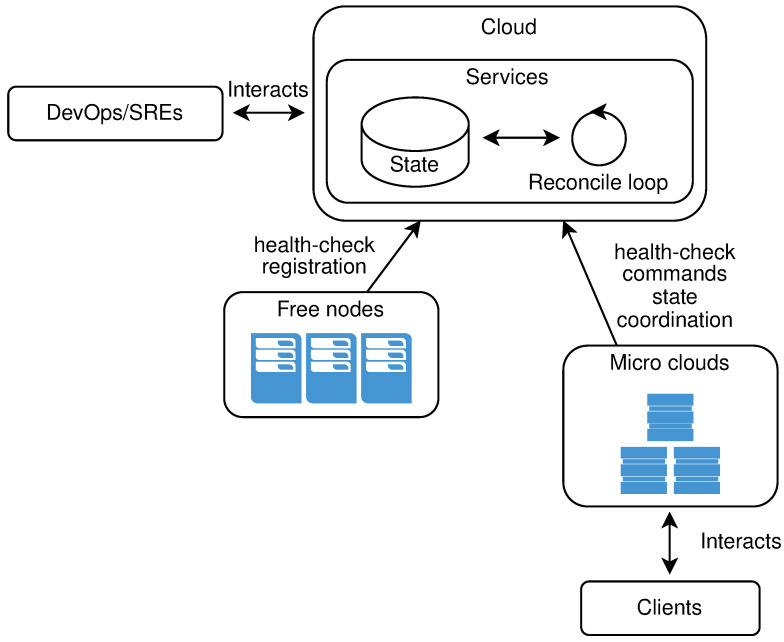
High level architecture of the system.

Figure 3 shows the high level architecture of the system.

The node can send its state over existing channels e.g., health-check pings to minimize load and data transferred over the network, or a dedicated channel just for this purpose may exist.

**Figure 4:**
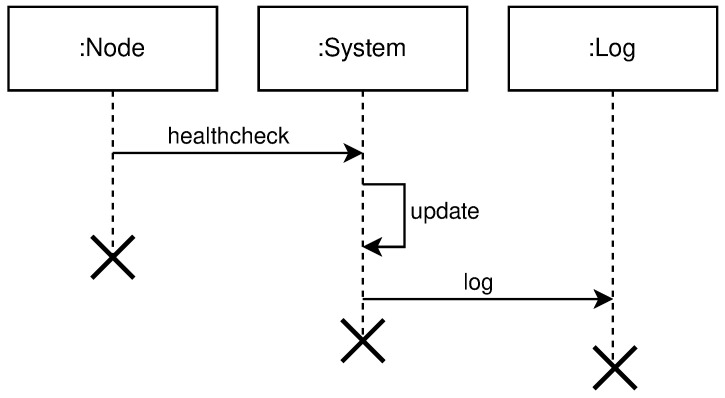
High level health-check protocol diagram.

Figure 4 shows the high level communication for health-check protocol.

If some node desires to be a part of the system, it must obey some simple rules:

- a node must run an operating system with a usable file system;
- a node must be able to run some isolation engine for applications, for example, containers or unikernels [38];
- a node must have available resources for utilization so that applications can be run or data stored;
- a node must have internet connection;
- a node must provide a list of attributes in the form of a list of key-value pairs—*labels*.

The design of rules is made simple intentionally, and could be helpful in certain situations. For example, if the number of requests suddenly increases (e.g., catastrophic event) beyond what currently available infrastructure can support, the system allows the volunteer nodes inclusion. These volunteer nodes can depreciate load for an indefinite period.

The concept of labels is based on the Kubernetes [39] labels mechanism, which is used as an elegant selecting and binding mechanism for its internal components. In μDCs labels can have all these roles, as well as a new role—presenting node attributes to the user (e.g., cpu: 4, mem: 100 GB, disk:ssd, storage: 120 GB, etc.). Labels represent a set of free-defined values, and they can also contain some node-specific attributes that should be pointed out (e.g., geolocation, architecture, os, etc.).

The user should submit a new state to the system in the form of a state description file—the desired state. When a new state description is accepted, the system will try to find the best possible pool of resources to use, or the exact pool of resources if available.

Here resources may be represented in three ways:

- Application resources like CPUs, GPUs, storage, network quotas, etc. if the user is submitting a new application into the cluster. For this type of task, virtualization will simplify resource management, and it will allow running applications over heterogeneous infrastructures;
- Infrastructure resources, if a user is creating a new clustered infrastructure. For this type of task, the user must specify what nodes are desired to be part of that cluster. Users can create dedicated clusters (e.g., processing, storage, etc.), or create clusters that can accept various types of tasks. Depending on the cluster type, nodes with different resources (e.g., CPUs, GPUs, storage, network. etc) can be targeted, forming a pool of available resources. This can be done by using some desired selector values that every node can have attached in the form of key-value pairs;
- Various configurations, The same system could be used for various configurations of nodes and clusters remotely. The model is easy to extend by just adding the new worker that will do a specific task when that kind of file is submitted to the system.

**Figure 5:**
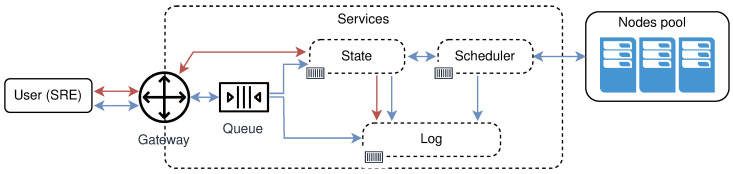
High overview of the services model.

All resources, whether application resources, infrastructure resources, or various configurations should be viewed as being part of sharable resource pools that can be controlled, managed, or repurposed at any given point in time. The extension of such a system could be as simple as adding a new service dealing with just that resource. Figure 5 shows a high overview of the services involved in all operations done by site reliability engineers (SREs) on the μCs infrastructure and/or free nodes. Blue arrows in the image symbolize the operations that will change the current state of the system. Red arrows represent read-only operations not changing the state of the system.

**Figure 6:**
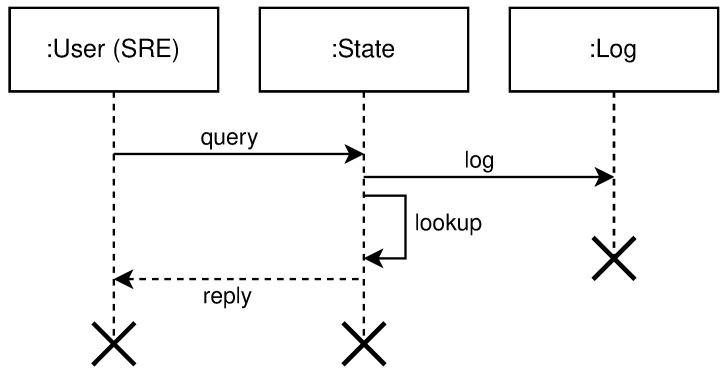
High level query operation diagram.

**Figure 7:**
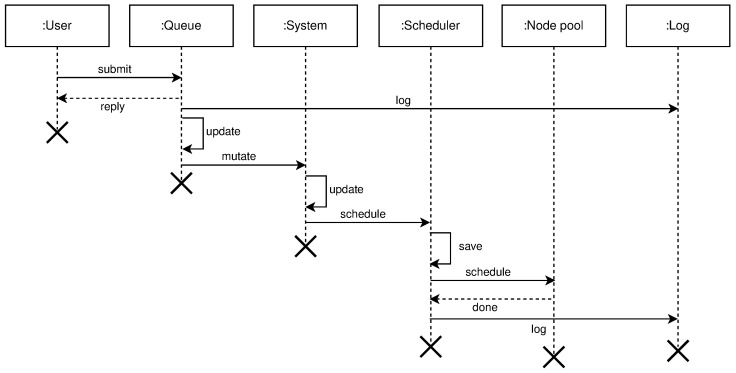
High level mutate operation diagram.

**Figure 8:**
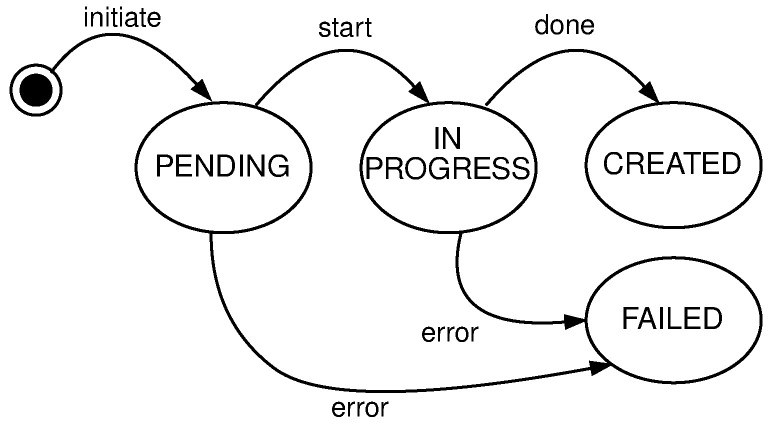
Newly submitted task state diagram.

**Figure 9:**
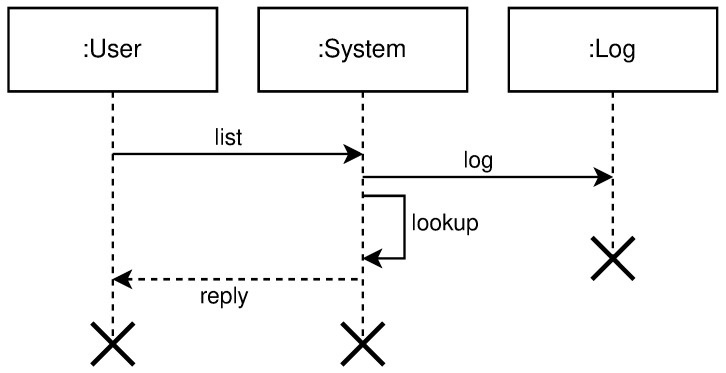
High level list operation diagram.

Three main operations that it should provide are:

- *query* free nodes for the purpose to create clusters, regions, and topologies. This operation involves the following entities: User, System, and Log. The user submits a selector that is a list of key-value pairs to the system. These values represent desired properties of the nodes the user is searching for. After receiving the selector list, the system will query its local register to compare selector with labels for every registered node in the system not used in some cluster—free nodes. The system will log all interactions with the user. Figure 6 shows the high level communication for query operation;
- *mutate* or creation of new clusters, regions, and topologies from the existing pool of free nodes (resources). Mutate operation requires following entities: User, Queue, System, Scheduler, Node Pool, and Log. The user submits a description file containing an infrastructure set up—a new infrastructure configuration. The queue accepts this new state and replies with an acceptance message to the user. The queue is drained at some configurable time *t*, to prevent overflow of the system, and it sends the mutate message to the system. The system accepts the *mutate* message, reserves nodes, and creates a new infrastructure configuration. When data is successfully stored, the system will schedule a new execution task, a new infrastructure to be physically set up. The scheduler executes the task and informs the node pool that needs to be part of the same cluster to start the membership protocol. When the membership protocol is done, the message is sent to both scheduler and the system. The system receives the health-check messages and properties of the cluster. The scheduler receives the done message, to signal that the scheduled task is done. Figure 7 shows the high level communication for mutate operation;
  When a user submits the *cluster formation message*, the system will accept the message and register the task with *PENDING* state. If the system cannot proceed further, for whatever reason (e.g., no available resources or nodes, etc.) the task is finished, and it goes to *FAILED* state, and this concludes the transaction. Otherwise, if there are no errors, and the system can proceed with the cluster formation protocol, the task will go to *IN PROGRESS* state.
  In this state, the system needs to save newly formed cluster information, prepare metrics service, add watchers for the cluster nodes health-check, etc. This operation spans multiple services, creating sub-transactions. The task state will prevent the users from applying other tasks, configurations, and actions on a not yet formed cluster. We can always invoke the rollback mechanism if any error happens during this process. Other options would be to try to fix the occurred issue with some of the retry strategies.
  If there are no errors, the cluster formation transaction finishes, changing the task state to *CREATED*. If there are errors during this process, the cluster formation transaction ends without creating the cluster. The task will go again to *FAILED* state, and this concludes the transaction. Figure 8 shows state diagram changes for the newly submitted task.
- *list* shows details about various parameters (e.g., nodes in clusters, regions, topologies, resource utilization, running applications, etc.). The entities involved in list operation are: User, System, and Log. The user submits what cluster/region/topology he wants details. The system will do a lookup on its state based on the query provided by the user. If such a record exists, it will show details about it (e.g., number of nodes per cluster, clusters per region, regions per topology, utilization, running applications, logs, etc.). All user interactions with the system will be logged. Figure 9 shows the high level communication for list operation;

All communications in this multi-tier infrastructure model should be independent of the model. We can implement all communication in the system using standard protocols (e.g., TCP, UDP, HTTP, MQTT, etc.). The model proposes that parts of the system communicate but should not impose communication protocols. The custom or proprietary protocols may be used.

### 3.3. User Data in Micro Clouds

μCs benefit from data locality and serving user requests from the best possible location—the closest μC to the user. They rely on the traditional clouds’ excessive capacities to store data, which is overwhelming for them. Hence μCs should store the fresh, most recently used data, and this is determined by the size of the μC given in Equation (1).

With this pattern in μCs, our solution meets two challenges:

- when a user moves to another place (e.g., from city to city, country to country), does the user data follow the user somehow, or should it be stationary? The decision should be on developers, users, and μC providers to decide—it depends on the service and type of data. The model anticipates different policies applied to the μC for every individual user or group of users—data plan. For example, if a user goes to another location and requests are served from another μC, the model can use a traditional cloud to locate the requested data. We can transfer requested data to the μC that is serving that request. The process is similar to the content delivery networks on the edge [23]. To minimize the network pressure, transfers should be only the requested data. An alternative option would be to use the traditional cloud as a backbone to serve user data if he moves to another location.
- how long should the user data be present in the μC, assuming that μCs need to serve many users, and they have limited resources. The model we propose relies on different policies applied to the user’s data. Depending on where the size of the μC lies in the specter given by Equation (1), μC providers and developers may offer different policies—time to live (TTL) [40] how long to store the data similar to the leases mechanism in cache systems [41].

The data retention and size in the μC are decided and optimized with (1) and (2), for the single user or group of users.

## 4. Deployment Properties

This section describes different aspects during the deployment of infrastructure. It presents problems and various deployment models to resolve these problems. It describes roles involved during the deployment in the μCs environment, and presents a proof of concept solution based on previously described model and deployment properties, tested in laboratory conditions.

### 4.1. Deployment Models

μC infrastructure deployment will not happen until the process is trivial [16]. The key to success is to simplify μC management. The problem is to decouple application management from the network and security [2]. Infrastructure and applications deployment in such a complex environment as μCs and three-tier infrastructure can determine many parameters.

How existing strategies handle the changes on the existing infrastructure or applications can be explored on [42]:

- A mutable deployment model is a model where changes are in place. In place, change means the existing infrastructure or applications get updated or changed during an update. This strategy is prone to leaving the system in an inconsistent state due to:
  − increased risk, because in-place change may not finish, which puts infrastructure or the application in a possible bad state. This is especially a problem if there are a lot of services and multiple copies of the same service because the same request may produce a different outcome. The possibility that the system is not available is a lot higher;
  − high complexity, this is a direct implication of the previous feature. Since the change might not get fully done, it cannot be guaranteed that the infrastructure or application is transitioned from one version to another — change is not discrete, but continues since we might end up in some state in between where we are now and where we want to be.
- An immutable deployment model is a model where no in-place changes on existing infrastructure or application are done whatsoever. In this model, the previous version is replaced completely with a new version that is updated or changed compared to the previous version. The previous version gets discarded in favor of the new version. When compared to the previous model, immutable deployment model:
  − has less risk, since the existing infrastructure or the application is not changed, but a new one is started and the previous one is shut down. This is important especially in distributed systems (DS) where everything can fail at any time;
  − has less complexity than the mutable deployment model. This is a direct implication of the previous feature since the previous version is shut down and fully replaced with the new one. This is a discrete version change and atomic deployment with deferring deployments with fast rollback and recovery processes;
  − requires more resources [43], since both versions must be present on the node for this process to be done. The second problem is the data that the application has generated should not be lost. The problem is solved by externalizing the data. We should not rely on local storage but store that data elsewhere, especially when the parts of the system are volatile and changed often. The key advantage of this approach is avoiding downtime experienced by the end-user when new features are released.

**Figure 10:**
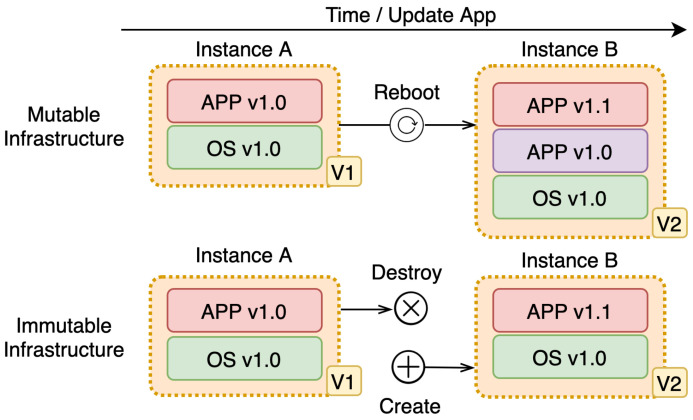
Difference between mutable and immutable deployment models.

Figure 10 summarizes the difference between mutable and immutable deployment models.

Immutability is a simple concept to understand, and it simplifies deployments. In distributed systems [43] where the state is not in the single place while nodes fail often, this is especially important.

In the three-tier infrastructure, we want to set up geo-distributed μC infrastructure as fast and as safely as possible to avoid application breakdown and degradation of QoS [2]. SREs define infrastructure on one end, while users expect that same infrastructure to be available on the other end, which involves a lot of communication and coordination. The system should embrace the failure that may occur and deal with it in some way. The easiest strategy is to accept discrete change—immutable deployment from one configuration to another. Write down some data, and ensure that it never changes. It can never be modified, updated, or deleted [44].

Immutable deployments help to fight the configuration drift problem, decreasing the overall system downtime. With the arrival of containers, change management in complex environments such as distributed systems may be as simple as stopping the previous version and starting the new one. This technique offers several benefits for deploying changes, offering various strategies for different situations. These strategies include:

- Blue-Green deployment, this strategy requires two separate environments: (1) *Blue* current running version, and (2) *Green* is the new version that needs to be deployed. When there is satisfaction that the green version is working properly, the traffic can be gradually rerouted from the old environment to the new one, for example by modifying the Domain Name System (DNS). This strategy offers near-zero downtime;
- A canary update is a strategy where a small subset of requests is directed to the new version—the canary, and the rest of them are directed to an old version. If the change is satisfactory, the number of requests can be increased, and it should be monitored how the service is working with increasing load, if there are errors, etc.;
- Rolling update strategy updates large environments, a few nodes at the time. The setup is similar to blue-green deployment, but here there is a single environment. With this strategy, the new version gradually replaces the old one. If for whatever reason the new version is not working properly on the larger number of nodes, rolling back to the previous version can always be done.

Highly available systems need to cope with hardware and software failures. Upgrading the software while the same software is running is not a trivial task to implement [45]. A rolling upgrade offers an upgrade of software without a noticeable downtime or other disruption of service. This is important when we want to update, upgrade or extend the infrastructure, but to sustain QoS.

**Figure 11:**
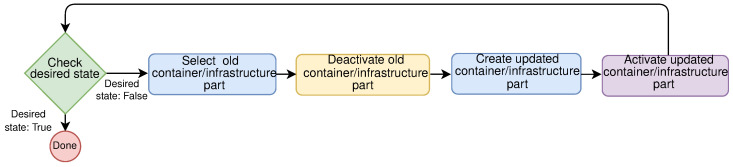
Rolling update flow chart.

For example, the rolling updates may change parts of the OS and services that run inside the containers. With the introduction of *LinuxKit*, Linux OS subsystems may be composed of very secure containers. As a result, systems created with LinuxKit have a smaller attack surface [46] than general-purpose systems. Ayres et al. [47] demonstrate how containers can promote efficient software updates using rolling updates in embed space. In the μC environment, this is especially important because of various heterogeneous nodes. When performing a rolling update, we can update all nodes seamlessly. It is a gradual process that allows users to update their infrastructure with only a minor effect on performance and no downtime. Figure 11 shows flow chart for rolling update in μCs.

Such update would be hard to implement using mutable model, since the transitions between states is not discrete, it might end up in some mid-state leaving the system in inconsistent state. As a result, it might get different response from the same service.

### 4.2. Deployment Roles

In our modern world of interconnected applications, there are several distinctive deployment roles. Each of them plays an important part so that modern software runs smoothly, and with less downtime. These roles could be summarized into a two categories:

- DevOps Engineers are in charge of a multidisciplinary organizational effort to automate application deployments through continuous delivery of new software updates [48]. DevOps combine software development with technology operations [49] to shorten the development life cycle.
- Site Reliability Engineers (SREs) are responsible for availability, latency, performance, efficiency, change management, monitoring, emergency response, and capacity planning [50]. It is a software engineering role and needs to have an understanding of the fundamentals of computing [51], applied to the infrastructure and operations problems.

DevOps engineers and SREs seem to be very similar roles, they are both trying to bridge the gap between development and operations. As such they have a very large conceptual overlap in how they operate [52], but also have some differences. Table 2 sums the differences between the DevOps engineers and SREs.

In a μC environment, both roles play a very important part so that the whole system stays up and running. DevOps engineers should be in charge of the execution of the application on the infrastructure (e.g., deployment, monitoring, scaling, QA), and SREs should deal with all bits and pieces of programmable infrastructure (e.g., forming, monitoring). Their job should focus on repurposing infrastructure resources and deal with infrastructure uptime. This approach removes entangling the geo-distribution concerns in the business logic of the application, deployment, and infrastructure [8].

The solution we propose is more towards SREs oriented. They are the ones dealing with pools of resources and programmable infrastructure. Their responsibility would be organizing and repurposing μC infrastructure, as well as monitoring and managing remote configuration and uptime.

When SREs prepare μC infrastructure—optimally organize a pool of resources to serve user requests locally first, and monitor infrastructure metrics [53], then DevOps engineers may set up their infrastructure for continuous delivery, application metrics, etc. to deploy services and applications that users may utilize.

### 4.3. Proof of Concept

The focus of this paper is not on the implementation details but presenting the possibility of the formation of disposable μCs at the edge dynamically using infrastructure as software principles. Therefore we are venturing into infrastructure programming at the edge. However to test its implementation based on the proposed model is possible—we implemented a proof of concept solution and tested it in laboratory conditions. Since laboratory conditions are significantly different from real-world scenarios, we did not analyze metrics (e.g., performance and network overheads or scalability aspects).

Based on the previously defined health-check protocol, list, mutate, query operations, and formal models defined in [19], we have implemented a proof of concept solution able to dynamically form disposable micro clouds, abstracting infrastructure to the level of software in laboratory conditions.

The proof of concept solution is implemented based on the referent architecture from Figure 3 and Figure 5, in a microservice manner using the Go programming language. As service to service communication, we used the gRPC framework, and services communicate using HTTP2 binary protocol. For the configuration store, we used an etcd, a strongly consistent, distributed key-value store. All physical nodes communicate with the rest of the infrastructure using the publish-subscribe principles. System to node communications and vice-versa is implemented using the messaging system NATS. All used frameworks are open-source.

The new state in *YAML* format is submitted to the system queue, where users specify what nodes need to form clusters, regions, and topologies. Users are allowed to override existing default labels for every node. They can also assign new ones on every node, region, or topology. Once submitted, the new state description goes over a few changes following the state diagram shown in Figure 8. When a new state is successfully stored in the system, the scheduler service will receive an event, to push changes to nodes about the cluster, region, or topology formation. Upon cluster formation message, nodes start group membership protocol to detect failures and disseminate the information through the network. For this purpose, we used a scalable weakly consistent infection-style (SWIM) protocol [54]. The messages in the cluster flow over standard UDP/IP.

All communication to the system is over the command-line interface (CLI) program. CLI communicates over HTTP protocol with the system gateway submitting JSON messages. This allows a dashboard web interface in the future.

Laboratory experiments are done on the twelve ARM physical nodes. Due to the lack of physical node numbers, we ran virtual nodes in containers on the physical nodes to increase network complexity. For every physical node, we started *n* virtual nodes, depending on the available resources on the physical node, running as Docker containers. This allowed us to increase network complexity by n-fold. One constraint is added to the automated experiment scripts—virtual nodes running on the same physical node cannot form clusters. Every virtual node gate a different set of test labels to meet the given constraint. When the test user is querying for the free nodes, he cannot see virtual nodes running on the same physical node.

We plan to analyze different metrics as part of our future work in real-world scenarios (e.g., power grid balancing, area traffic routing, and control, etc.).

## 5. Proposed Model Case Study

This section explores the case study of a proposed model. Section 5.1 presents example usage of the proposed model in case of COVID-19 area traffic control. The Section 5.2 compares the proposed model with the existing similar models, advantages and shortcomings.

### 5.1. COVID-19 Area Traffic Control

Let us consider a scenario where some city has an up and running micro cloud environment in its area. Here city topology can be formed, where the whole city represents one topology. Regions can be organized as needed, but for the sake of example we can follow the natural subdivision of the city onto municipalities, where every municipality represents one region inside the city topology. Depending on the population density, we can organize clusters inside the region as needed. Some regions may have more clusters, while others may have fewer.

If COVID-19 suddenly hits that city, an increasing number of ambulance vehicles needs to be routed to the hospitals fast. Developers can create the application running inside a micro cloud—frontend service that will track the actual position of these vehicles within the street grid, process that information, and in combination with another - traffic control service, usually managed by the appropriate city agency, give these vehicles dynamic priority. Combined, those two services can calculate or predict traffic jams, and the traffic control service can “clear the path”—closing the traffic lights on cross streets prior to emergency vehicle arrival at the crossroads, and prolong open intervals along the vehicle calculated route to increase traffic flow. With such interaction, these vehicles could be routed faster and safer to the nearest hospitals. Ambulance vehicles may monitor patient health state on the way to the hospital, transfering that data to the third frontend service and eventually to electronic health record—backend service, giving medical personnel much needed information before the patient arrives. Additionally, if the patient state suddenly changes, it can provide rerouting to different hospitals if needed. Since such a system would rely on live traffic information gathered through interaction of sensor nodes in specific region and traffic control endpoints running on some nodes—it would also provide fast recovery of traffic to normal flow after the emergency vehicle passes some points in the street grid—therefore avoiding prolonged traffic jams which could hamper other emergency vehicles movement. It is important to notice that the calculation and management of the route is lowered to the level of the region, which provides a faster response to real conditions and less disruption of traffic outside the zone in which the vehicle is. The fourth frontend service may collect depersonalized data in real-time, preprocess and transfer to the cloud for deeper analysis—backend service. This strategy may help researchers to better understand and fight the disease.

Applications doing these tasks will require more resources in such scenarios in order to work properly. For this, we may choose to organize clusters inside regions in a different way. A few clusters may even be dedicated just for this purpose, while others will serve the rest of the running applications. Even regions could be joined into bigger regions providing even more available resources. All this could be done dynamically with the proposed model, and descriptively.

After the end of the outbreak, when everything is back to normal, the resources can be reorganized as before. If the outbreak returns, they can again be reorganized in the same way or using some better strategy. It is important to notice how we can elastically reorganize resources as needed ad hoc, without knowing future scenarios.

**Figure 12:**
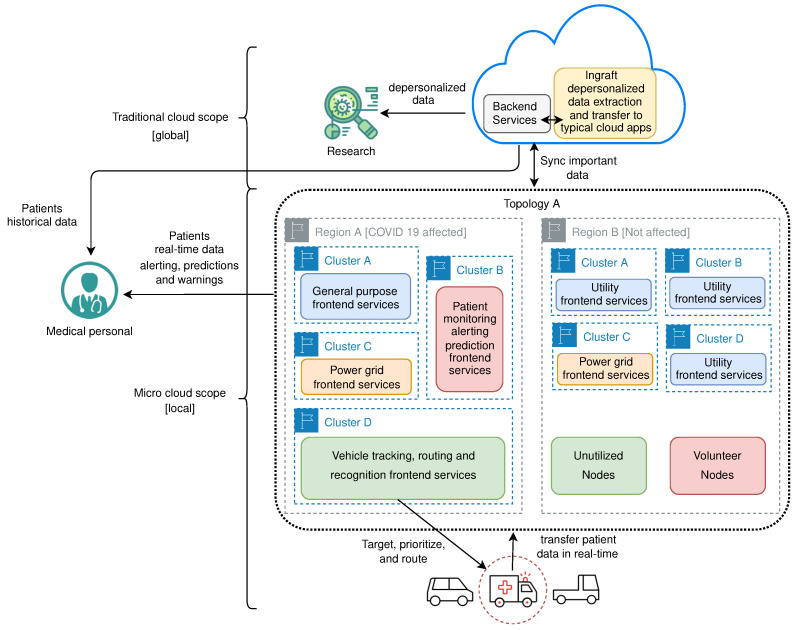
Conceptual architecture model for COVID-19 area traffic control example.

Figure 12 depicts previously described example, trough conceptual architecture model.

### 5.2. Discussion

Proposed model allows organization and reorganization of resources as needed dynamically and elastically in a similar way the cloud does. This feature allows users to develop applications without some specialized infrastructure for different types of applications. The system accepts local requests, even if the cloud is not reachable, making it more robust in terms of network failures [1], giving the users an illusion that the cloud is closer to them. This minimizes the potentially huge round-trip time of the cloud [55].

Compared to the similar existing models [56,57,58,59,60], our model offers few benefits and few short shortcomings.

The main strength of the proposed model allows developing a vider range of applications without the need for specialized hardware or software. This allows users to build their applications, similarly as they would build them for the cloud. Some specialized models require specialized infrastructure in order to resolve a single problem.

On the other hand, these specialized models run optimized versions of applications developed to use maximum of the underneath hardware and software. As such, they might outperform the proposed model in terms of speed. On the contrary, our model offers much more development freedom for the users, in terms of agility and applicability.

This allow organization of storage and processing resources according to priority. The entire state can organize its resources and completely manage its digital infrastructure, creating applications market that will help its citizens.

## 6. Conclusions

This paper presents a possible solution to dynamically manage infrastructure in μDCs at the edge in a multi-tier environment where we have clients at the bottom, μCs in the middle, and a cloud environment at the top. This multi-tier infrastructure supports data processing locally but also uses huge availability of cloud resources when necessary.

We have used infrastructure as a software principle to abstract infrastructure at the edge to the software level, using familiar, already existing, tools, best practices, versioning, etc. for software development, to support future real-time applications.

The model of μDCs we present is influenced by the cloud computing infrastructure model, but adapted for a different environment. This gives us the ability to organize nodes dynamically into clusters, regions, and topologies to form μCs at the edge, providing resources at the best place they are needed. Our model allows coverage of the arbitrarily vast geographic region, with the ability to descriptively organize, reorganize, and repurpose the infrastructure resources as needed where the size of the infrastructure resource pool is determined by the population’s needs.

When more resources are needed (e.g., to support bursts of requests, catastrophic events, etc.), our model can extend clusters with additional nodes. When these resources are not needed anymore, our model can release them back to a pool of free resources, similar to the cloud model. One possible drawback of our model is that initial investments may be high, but cloud providers or government authorities may deploy infrastructure and lease it to users in the familiar pay-as-you-go model, already used in the cloud.

First, we have introduced infrastructure as a software principle, and how infrastructure could be treated in the same way as software, and the benefits of such an approach. Next, We have described a design of a possible solution based on the existing models widely adopted in the cloud for infrastructure deployment, with different development roles that could be used while developing applications that could run in such multi-tier infrastructure. We have also argued about the importance of formal models, and their benefits in such a complex multi-tier environment, where applications span over clouds and μDCs.

As part of our future work, we are planning to test the proof of concept implementation in some real-world geo-distributed environments (e.g., measurements of different parameters relevant to detect hazardous occurrences, real-time detection, and alerting of changes in air quality essential for lung patients, management in power grids, etc.) to analyze the performance, network overheads, and scalability aspects of the proposed model.

We are planning to extend our system with namespaces allowing multi-tenancy in the system. Namespaces would provide the creation of the virtual clusters, running on the same physical hardware. Additionally, we are planning to add remote management, where users can disseminate configurations, security credentials, and actions over nodes in one or multiple clusters. We are planning to develop a prototype based on the proposed model.

## Figures and Tables

**Table 1:** Comparison between traditional cloud and micro cloud infrastructure.

Attribute/Cloud Type	Traditional Cloud	Micro Cloud
Logical	Cloud Provider	Topology
		Region
Physical	Region	Cluster
	Zone	

**Table 2:** The differences between the DevOps and SREs roles.

Feature	DevOps	SREs
Task	Scaling, uptime, robustness	Development pipeline
Essence	Practices and metrics	Mindset and culture
Team structure	Wide range of roles: QA, developers, SREs etc.	SREs with operations and development skills
Focus	Development and delivery continuity	System availability and reliability
Goal	Bridge the gap between development and operation	

## Data Availability

Data sharing not applicable.

## Abbreviations

The following abbreviations are used in this manuscript:

DS	Distributed systems
CC	Cloud computing
ECC	Edge-centric computing
IaC	Infrastructure as code
IaS	Infrastructure as software
IoT	Internet of things
DC	Data center
DNS	Domain Name System
DSL	Domain-specific language
SRE	Site Reliability Engineer
VM	Virtual machine
OS	Operating system
μDC	Micro data center
μC	Micro cloud
μCc	Micro cloud cluster
IaaS	Infrastructure as a service
